# Ensemble Framework of Deep CNNs for Diabetic Retinopathy Detection

**DOI:** 10.1155/2020/8864698

**Published:** 2020-12-09

**Authors:** Gao Jinfeng, Sehrish Qummar, Zhang Junming, Yao Ruxian, Fiaz Gul Khan

**Affiliations:** ^1^College of Information Engineering, Huanghuai University, Zhumadian, Henan 463000, China; ^2^Henan Key Laboratory of Smart Lighting, Zhumadian, Henan 463000, China; ^3^Department of Computer Science, COMSATS University Islamabad, Abbottabad Campus, Islamabad, Pakistan; ^4^Henan Joint International Research Laboratory of Behavior Optimization Control for Smart Robots, Zhumadian, Henan 463000, China

## Abstract

Diabetic retinopathy (DR) is an eye disease that damages the blood vessels of the eye. DR causes blurred vision or it may lead to blindness if it is not detected in early stages. DR has five stages, i.e., 0 normal, 1 mild, 2 moderate, 3 severe, and 4 PDR. Conventionally, many hand-on projects of computer vision have been applied to detect DR but cannot code the intricate underlying features. Therefore, they result in poor classification of DR stages, particularly for early stages. In this research, two deep CNN models were proposed with an ensemble technique to detect all the stages of DR by using balanced and imbalanced datasets. The models were trained with Kaggle dataset on a high-end Graphical Processing data. Balanced dataset was used to train both models, and we test these models with balanced and imbalanced datasets. The result shows that the proposed models detect all the stages of DR unlike the current methods and perform better compared to state-of-the-art methods on the same Kaggle dataset.

## 1. Introduction

**Figure 1:**
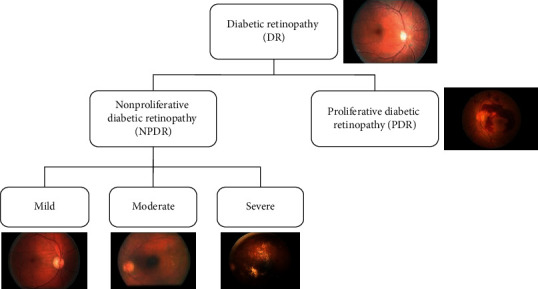
Stages of DR.

Diabetes mellitus, commonly known as diabetes, causes high blood sugar. Persistently high blood sugar level leads to various complications and general vascular deterioration of the heart, eyes, kidneys, and nerves [1]. Diabetic retinopathy (DR) is one of the leading diseases caused by diabetes [2]. It damages the blood vessels of the retina, for those who have diabetes type-I or type-II. DR is classified into two major classes: nonproliferative (NPDR) and proliferative (PDR) [3]. In NPDR, the changes are detected in the retina that needs to be monitored. The NPDR is subdivided into three stages according to the level of damage in the retina, namely, mild, moderate, and severe. The NPDR would turn to PDR with a high risk if timely not diagnosed. In PDR, fragile (breakable) new blood vessels form on the surface of the retina over time. These abnormal vessels can bleed or develop scar tissue, causing severe loss of sight (neovascularization, vitreous hemorrhages). The disease progresses from mild NPDR to PDR, as shown in Figure 1. The influence of DR can be alleviated if it can be detected and treated at an early stage.

Globally, patients are expected to increase from 382 million to 592 million by 2025 with diabetes [4]. And, with DR, this is excepted to increase from 126.6 million to 191.0 million by 2030 [5]. The international diabetes federation estimates that global incidence of adult diabetes will be increased from 8.4% in 2017 to 9.9% by 2045 [6]. In the early stage, patients are asymptomatic but in advanced stage, it may lead to blurred vision, floaters, and visual acuity loss. Hence, it is difficult and utmost important to detect DR in early stages to avoid the worse effect on later stages.

Figure 1 shows the images of all stages; it is clear that normal and mild stage visually look similar. So, it is difficult to detect mild stage. The color fundus images are used to diagnose DR. The manual analysis can only be done by experts, which is expensive in terms of time and cost. Therefore, it is important to use computer vision techniques to automatically analyze fundus images. Many automatic techniques have been applied to detect DR. Computer vision-based method can be divided into two categories: hand-on engineering [7] and end-to-end learning [8, 9]. The hand-on engineering methods were based on single selected features, such as blood vessels outline, exudes, hemorrhages, microaneurysms, and maculopathy of retinal fundus image or their combinations [7]. The end-to-end learning automatically learns features and hence performs better classification. Many hand-on engineering and end-to-end learning methods [8–14] detect DR using Kaggle dataset, but no approach could detect mild stage. To treat this fatal disease, early stage detection is important. This study focuses on detecting all the stages of DR (including mild stage) using end-to-end deep ensemble networks. The result shows that the proposed approach outperforms state-of-the-art methods.

The remainder of this paper is organized as follows.  Section 2 reviews recent literature related to DR detection. The proposed model is described in Section 3. The performance evaluation and results are presented in Section 4. Conclusions of the research and suggestions for future work are summarized in Section 5.

## 2. Literature Review

The classification of DR has been extensively studied in the literature. Several studies have proposed methods to detect DR stages and its severity [15–21]. DR can be detected in many ways such as single stage detection and binary classification. The problem in these methods is that we cannot detect the severity of the disease. So, the solution is multiclass classification. Pratt et al. [20] proposed a CNN-based method; however, Pratt et al.'s [20] architecture did not detect the mild stage and achieved 30% sensitivity and 95% specificity, which indicates that the architecture did not classify the affected stages properly. The major issue with their sensitivity results is that they used an imbalanced/skewed dataset. Another study [22] shows a better result when using a balanced dataset for training; however, a balanced dataset has not yet been used to detect DR when testing a model. In addition, Bravo et al. [23] used a balanced dataset to train a model and achieved 50.5% accuracy; however, the test dataset was not balanced. Further, Chandrakumar et al. [16] implemented a deep CNN deployed with a dropout layer to detect DR and achieved 94% accuracy on the DRIVE dataset. They used spatial feature analysis for detection; however, the number of samples in the dataset was not sufficient. Furthermore, Takahashi et al. [21] proposed an AI disease-staging system that grades DR by using a retinal area. That proposed system directly suggests treatments and determines prognoses. However, they used modified Davis staging, which is not commonly employed for grading DR. In that study, in the network misclassified some images, the false negative rate was lower than the false positive rate.

Moreover, deep learning classification algorithms have proven to be very effective if the model is trained in a supervised manner with sufficient data. Transfer learning with different CNN models has achieved good accuracy and DR classification results [24, 25]. Kori et al. [26] used an ensemble technique to detect DR stages and DME, and, according to [27], ensemble models perform well. Similarly, Choi et al. [28] pretrained a model with transfer learning and used an ensemble voting technique that improved accuracy. Hagos et al. [29] used transfer learning to train an Inception-V3 model that classified all stages. They achieved 90.9% accuracy on the test dataset. Similarly, Carson et al. [30] also used transfer learning to classify all DR stages. However, they all use transfer learning and an ensemble technique but did not use balance dataset to train and test a model.

The literature shows that researchers have applied or proposed various methods for detecting and classifying DR stages. As mentioned above, there are many ways to detect DR, but multiclassification detects the severity of DR stages. In the multiclass classification, the stages are divided into 5 stages as discussed in the Introduction section. However, most of the literature is not able to properly classify all stages of DR, especially the initial stage. It is important to detect the early stage of DR for treatment, as at a later stage it is difficult to cure and can lead to blindness. To the best of our knowledge, no other work has identified the early stages using the Kaggle dataset we used for our research with a balanced dataset. Our models can detect the mild stage and perform better than the current state of the art. Moreover, in the literature, no one has shown the result of a balanced dataset. Imbalanced dataset can lead to bias in classification accuracy. If the samples in the classes are equally distributed in a balanced dataset, then the network can learn the possibilities correctly, but, in the case of an imbalanced dataset, the networks exceed the high sampling class.

## 3. The Proposed Method

### 3.1. Preprocessing

**Figure 2:**
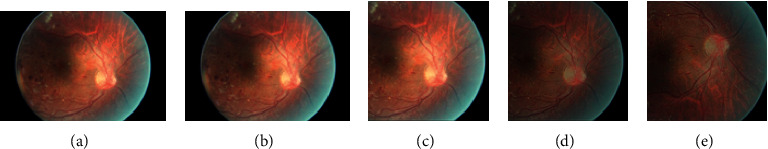
Preprocessing steps: (a) original image, (b) aspect ratio, (c) cropped 512 × 512 image, (d) normalization applied to cropped image, and (e) augmented image.

The different preprocessing steps we perform on the input dataset before giving it to the model are shown in Figure 2.

We used the Kaggle dataset which contains 35126 color fundus images, each of size 3888 × 2952 pixels, shown in Figure 2(a). It contains an image of five classes according to DR severity.  Table 1 shows the distribution of sample images in different classes of Kaggle dataset. The distribution of samples images is shown in the first row of Table 1, which is clearly imbalanced. Training a deep network with imbalanced dataset may lead to biasness of classification. In the first preprocessing step, we resized each image to 786 × 512, which maintains the original aspect ratio, shown in Figure 2(b). Moreover, we randomly cropped 512 × 512 patches to reduce training overhead, as depicted in Figure 2(c). Furthermore, to speed up training time and avoid feature biasness, each image was mean normalized as shown in Figure 2(d). In the end, the dataset is balanced with upsampling [22]. Upsampling was performed by rotating an image to 90 degrees, augmenting minority classes, and flipping image, as shown in Figure 2(e), which increase the size of dataset, balance the samples in each class, and avoid overfilling. The distribution of balanced samples images of the different classes is shown in second row of Table 1, in which the balanced dataset is divided into three sets: training (64%), testing (20%), and validation (16%), and the validation set is used during the training to check and reduce overfitting. Finally, we make batches from the training dataset, i.e., dataset_1, dataset_2, and dataset_3, which are used to train the models.

Table 1 shows the total number of samples in each class. The original dataset contains 35126 samples with different numbers of samples in each class; i.e., this dataset is highly imbalanced. To balance this dataset, we performed the preprocessing steps mentioned above. The balanced dataset contains 129050 samples. This dataset is further divided into the training, test, and valid datasets, and the training dataset is further divided into three smaller datasets, dataset_1, dataset_2, and dataset_3, each of which has an equal number of samples, i.e., 27530.

### 3.2. Model 1

The combination of several machine learning techniques into a single predictive model is called an ensemble method. It may include decreasing variance (bagging) and bias (boosting) or improving prediction (stacking) [31]. We employ a bagging ensemble technique, wherever the bagging represents bootstrap aggregation. The variance of an estimate can be reduced by taking an average of multiple estimates. Bootstrap sampling is used in Bagging method to obtain data subsets for training a base learner. The output of a base learner is aggregated by voting and averaging for classification.

The proposed approach ensembles the result of three datasets trained with DenseNet-121.  Algorithm 1 presents the proposed model in detail. Let *ℋ* *=* *{DenseNet-121}* be a pretrained model. A model is fine-tuned with three fundus images datasets *(X,Y),* where *X* is the number of images, size of 512 × 512, and *Y* contains the corresponding labels; *Y* *=* *{y/y ϵ {normal, mild, moderate, severe, PDR}}*. Three bags of datasets are divided into mini batches, each of size *n* = 5, such that *(X*_*i*_*,Y*_*i*_*) ϵ (X*_train_*, Y*_train_), *i* = 1, 2,…, N/*n;* iteratively optimizing (fine-tuning) the CNN model *h ϵℋ* reduces the empirical loss:

(1) $$\begin{array}{c} L\left(w;X_{i}\right)=\frac{1}{n}\underset{x\epsilon X_{i},\;y\epsilon Y_{i}}{\sum }l\left(h\left(w,x\right),y\right), \end{array}$$

where *x* is the input, *y* is the class, *h(x, w)* is a CNN model predicting class *y* for input *x*, and *w* and *l* are the categorical cross-entropy penalty functions. The stochastic gradient descent is used to update the learning parameters:

(2) $$\begin{array}{c} w_{t+1}=\;\gamma w_{t}-\alpha \nabla_{w}J\left(x^{i},y^{i};w\right), \end{array}$$

where ***α*** is the learning rate and is set as 0.0001. ***γ*** is a Nesterov momentum which helps accelerate SGD in the relevant direction and dampens oscillations, set as 0.9. In the start *w*_*t*_, *t* = 0 is initialized to the learned weights of the model *h ϵℋ* using transfer learning. The output layer of a model, *h ϵℋ*, uses *SoftMax* as an activation function which generates the probabilities of how much the input belongs to the set of different classes *{normal, mild, moderate, severe, PDR}*. We use 50 epochs for training with early termination if the model starts overfitting.

**Figure 3:**
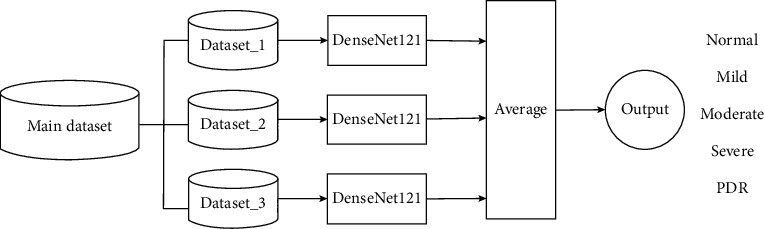
Ensemble Model 1 for classification of DR stages.

In case of testing a model, an unseen example from the class label is used to predict the model efficiency. The results of all models were combined by averaging which provides a unified output. The ensemble approach leads to better performance by combining the strengths of individual models. The proposed bagging ensemble is shown in Figure 3. Let *X*_test_ be a new test sample; then the ensemble output is given by

(3) $$\begin{array}{c} m^{*}=\;\arg\;\max_{m}\;\frac{\sum_{h\in Hu}h\left(w,X_{\text{test}}\right)}{\left|Hu\right|}, \end{array}$$

where *h(.)* is the fine-tuned model, |*ℋ*| is the cardinality of the models, and *m* represents the different modalities such that *m ϵ {normal, mild, moderate, severe, PDR}*.

### 3.3. Model 2

**Figure 4:**
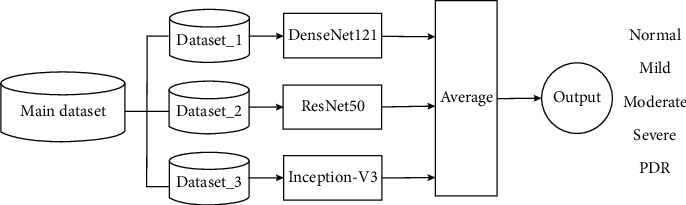
Ensemble Model 2 for classification of DR stages.

This model is an improved version for the classification of DR stages in this study.  Algorithm 2 presents the details of the proposed model. Let *ℋ* *=* *{DenseNet-121, ResNet50, Inception-V3}* be pretrained models. The models are fine-tuned with three fundus images datasets *(X,Y),* under the same conditions as Model 1 (Section 3.2). The proposed bagging ensemble for Model 2 is illustrated in Figure 4.

## 4. Results

In this section, the results of the proposed models are discussed using imbalanced and balanced datasets. The proposed models were trained utilizing a high-end Graphics Processing Unit (NVIDIA GeForce GTX 1070 Laptop) with the CUDA Deep Neural Network library. In addition, the TensorFlow and Keras (http://keras.io/) were used (Keras as deep learning package and TensorFlow as machine learning back end).

### 4.1. Performance Parameters

We used the following metrics to evaluate the performance of the proposed model. Here, the objective was to properly classify all DR stages specially the early stages of DR.

 
*Accuracy.* Accuracy can be calculated as positive and negative classes:

(4) $$\begin{array}{c} \text{accuracy}=\frac{\text{TP}+\text{TN}}{\text{TP}+\text{TN}+\text{FP}+\text{FN}}. \end{array}$$

  Here, TP is true positive, TN is true negative, FP is false positive, and FN is false negative.
 
*Recall/Sensitivity.* Recall (or sensitivity) is also known as the TP Rate (TPR).

(5) $$\begin{array}{c} \text{sensitivity}=\frac{\text{TP}}{\text{TP}+\text{FN}}. \end{array}$$

 
*Specificity.* Specificity is also known as the TN Rate.

(6) $$\begin{array}{c} \text{specificity}=\frac{\text{TN}}{\text{TN}+\text{FP}}. \end{array}$$

 
*Precision.* Precision is the rate of correctly predicted number of classes over the total number of classes predicted by the model.

(7) $$\begin{array}{c} \text{precision}=\frac{\text{TP}}{\text{TP}+\text{FP}}. \end{array}$$

Area under the Curve [32] and Receiver Operating Curve (AUC-ROC) [33] represent the degree or measures of separability of different classes. The higher the AUC score, the better the model and vice versa.

Table 2 shows the number of samples in the datasets. The imbalanced dataset contains 5608 samples, and the balanced dataset contains 25800 samples (5160 samples in each class). These samples are obtained by the preprocessing steps.

To show the effect of the imbalanced dataset, we have used two datasets: (i) imbalanced dataset and (ii) balanced dataset. In the end, we also have shown the comparative results of the models. The distribution of test dataset samples is given in Table 2.

### 4.2. Model 1

**Figure 5:**
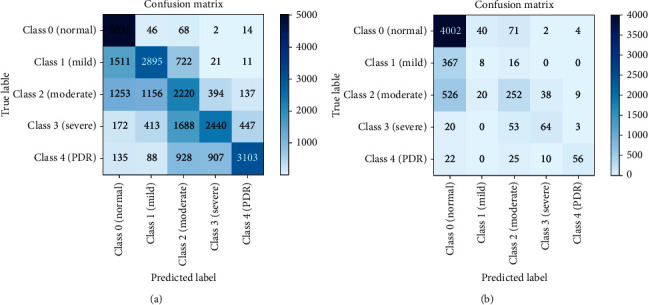
Confusion Matrices: (a) balanced dataset, (b) imbalanced dataset.

**Figure 6:**
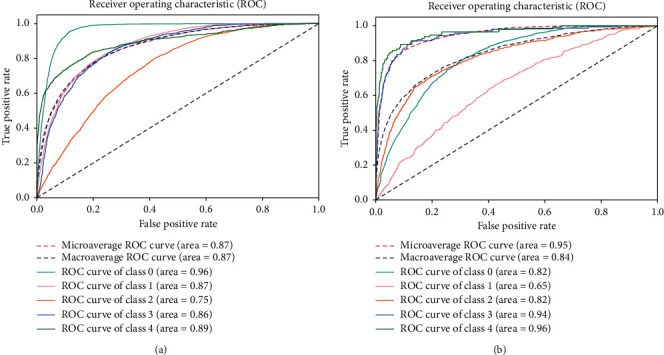
ROC for Model 1: (a) balanced dataset and (b) imbalanced dataset.

Model 1 is similar to a bagging technique where only a single base model is used with different bags of datasets. We consider the DenseNet-121 dataset as the base model, and we used three balanced datasets to train this model discussed in Section 3.1. The results were computed using a batch size of 5. The resulting confusion matrices are shown in Figure 5. Each class of a dataset is equally distributed in the balanced dataset; therefore, the classification result is also better than the result obtained using the imbalanced dataset. With the balanced dataset, Class 1 (mild) is predicted accurately compared to the imbalanced dataset where only eight images are predicted. We obtained higher accuracy with the imbalanced dataset due to the unequal distribution of samples. We can also see a difference in Class 4 (PDR) prediction. Here, the balanced dataset outperforms the imbalanced dataset. The overall accuracy achieved by this model was 78.13% and 60.80% on the imbalanced and balanced datasets, respectively. To obtain more accurate results, we also evaluated results using the ROC curve, shown in Figure 6, and class-wise results, given in Table 3.

Table 3 lists class-wise results for both balanced and imbalanced datasets in terms of recall, precision, and specificity. As can be seen, the results are significantly better in the balance dataset compared to those in the imbalanced dataset. The values differ due to the different number of samples. In the recall column, all class values for the balanced dataset are better than those for the imbalanced dataset, particularly for Class 1 and Class 4. These classes are predicted very well in Model 1. In the precision column, Class 1 and Class 4 are predicted more accurately in the balanced dataset than in the imbalanced dataset. In the specificity column, the values for the balanced dataset are less than the values for the imbalanced dataset. Balanced dataset results are better than imbalanced dataset results; however, the overall accuracy values differ. As accuracy results differ, we also find the ROC curve for the balanced and imbalanced dataset as shown in Figure 6.

The ROC curve for the balanced dataset indicates the equal distribution of samples. The most accurate prediction was obtained for Class 0 because its area is 0.96, which is near 1. We can also see that Classes 1-4 curves are also near 1, which means that Model 1 classifies all images accurately. With the imbalanced dataset, the highest curve is achieved by Class 4 because it has the lowest number of samples, and the predicted images are also high, which is why the curve is near 1. Class 0 has more samples than all other classes, and the predicted value is also very high. However, as the number of samples increases, the curve decreases to 0, which is why the area of Class 0 is 0.82.

### 4.3. Model 2

**Figure 7:**
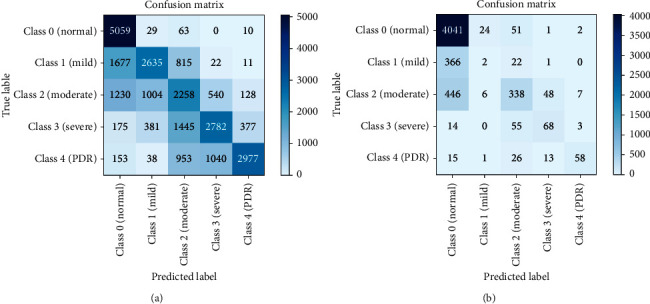
Confusion matrices: (a) balanced dataset and (b) imbalanced dataset.

Model 2 is the same as Model 1; however, the training models differ. With Model 2, we trained three deep CNN models, i.e., DenseNet-121, ResNet50, and Inception-V3, with different bags of training datasets. Note that the same test dataset was used for all three models. The models are tested with balanced and imbalanced datasets, and the results are shown in Figure 7. The classes are distributed equally in the balanced dataset; therefore, the classification result is better than that of the imbalanced dataset. With the balanced dataset, Class 1 (mild) is predicted accurately, while in the imbalanced dataset only two images are predicted. In addition, in the balanced dataset, approximately 2000 images are predicted accurately in each class; 5000 images were predicted for Class 0. We obtain higher accuracy with the imbalanced dataset due to the unequal sample distribution. We can also see a difference in Class 1 (mild) and Class 4 (PDR) predictions. For these classes, the balanced dataset outperforms the imbalanced dataset. The overall accuracy achieved by this model was 80.36% and 60.89% on the imbalanced and balanced dataset, respectively.

**Figure 8:**
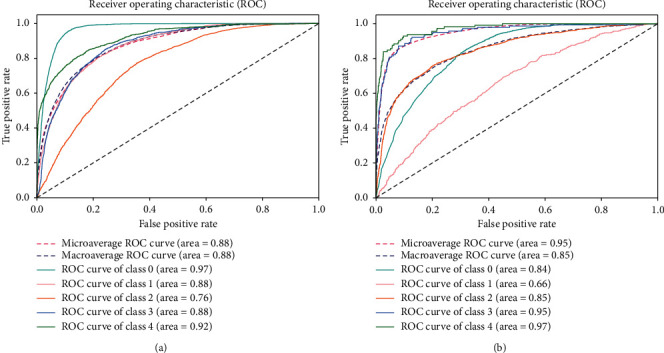
ROC curves for Model 2: (a) balanced dataset and (b) imbalanced dataset.

The ROC curves for the balanced and imbalanced datasets are shown in Figure 8. For more accurate results, we also consider class-wise results, as shown in Table 4. Table 4 shows the class-wise results for both balanced and imbalanced datasets in terms of recall, precision, and specificity. The balanced dataset shows improved results compared to the imbalanced dataset, in terms of recall, precision, specificity, and accuracy. In the recall and precision columns, all values were improved for the balanced dataset, and specificity was also improved. Overall accuracy for the imbalanced dataset was higher than that for the balanced dataset due to the unequal number of samples in the imbalanced dataset. The negative class (Class 0), which is predicted accurately, has the large number of samples; however, positive Classes1-4 are misclassified. We calculated ROC curves for both balanced and imbalanced datasets shown in Figure 8.

The ROC curve for the balanced dataset shows the equality in sample distribution. The more accurately predicted class is 0 because its area is 0.97, which is close to 1. We can also see that Classes 1- 4 curves are also near to 1, which means that Model 2 classified all images accurately. With the imbalanced dataset, the highest curve was achieved by Class 4 because it has lowest number of samples, and the predicted number of images was also high, which is why the curve is close to 1. There were more samples in Class 0 than the other classes, and the predicted value is also very high. However, as the number of samples increases, the curve decreases to 0, which is why the area of Class 0 is 0.84. It is vital to detect all DR stages for early treatment of the disease. Class 1 (mild) is the first stage of the disease and detection at this stage is important to provide better treatment.

### 4.4. Model Comparison

In this section, the results obtained in Model 1 and Model 2 are compared with each other and also to those of other models. Model 2 returned much better results than Model 1 because the stages are classified properly. Models 1 and 2 achieved 78.13% and 80.36% accuracy, respectively, as shown in Table 5. The models are also trained with different batch sizes, and accuracy, recall, and specificity results change as the batch size changes given in Table 5.

Various models are compared in Table 5. These models were trained with the same architecture but with different batch sizes, except for the model proposed by Pratt et al. [20]. Models batch_size_2, batch_size_3, and batch_size_4 were trained with Model 1 architecture but with different batch sizes, i.e., batch sizes 2, 3, and 4, respectively. Model 1 used batch size 5. The results for Model 1 with batch size 5 are discussed in detail in Section 4. DenseNet-121, ResNet50, Inception-V3, Xception, and Dense169 were trained using the entire balanced dataset with batch size 8, and these models are tested on an imbalanced dataset. Pratt et al.'s [20] model is used for comparison with the proposed models because that study [18] also used the Kaggle dataset and investigated classification of DR stages. Model 2 was trained with batch size 5, and those results are also discussed in Section 4. Our proposed models, 1 and 2, outperform Pratt et al.'s [20] model if we consider accuracy, recall, and specificity of the model tested on the imbalanced dataset. However, Model 2 outperforms both Model 1 and the Pratt et al.'s [20] model relative to recall and specificity.

**Figure 9:**
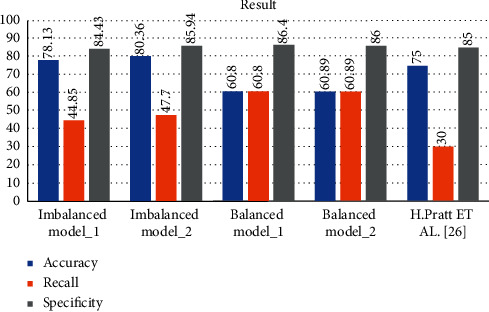
Comparison results of Model 1 and Model 2 for balanced and imbalanced datasets and Pratt et al.'s [20] model.

The results of Model 1, Model 2, and the model of Pratt et al. [20] are compared in Figure 9. Pratt et al.'s [20] model achieved a specificity value of 95%, which is a higher value than that achieved by both Model 1 and Model 2 with the imbalanced dataset. With the imbalanced dataset, Model 1 and Model 2 achieved 84.43% and 95.84%, respectively. Compared to Pratt et al.'s [20] model, recall and accuracy increased from 30% to 44.85% and 75% to 78.13%, respectively, with Model 1. With Model 2, recall was 47.7% and accuracy was 80.36%, which means that Model 2 yielded better classification than Pratt et al.'s [20] model and Model 1 and classified all stages of DR. Recall values are much higher in the balanced dataset compared to those in imbalanced dataset. Accuracy and recall can be improved if a larger batch size is used.

## 5. Conclusions

Diabetes is one of the fast-growing diseases in the world and causes many diseases. Diabetic retinopathy (DR) is one of those diseases. DR has different stages from mild to severe and then PDR (Proliferative Diabetic Retinopathy). In the later stages of the disease, it may lead to symptoms such as floaters, blurred vision, and finally a vision loss. Manually diagnosing this disease is tedious and error prone. So, computer vision-based techniques applied to diagnose a disease in an automatic way are discussed in the literature. In this study, we presented two deep ensemble CNN Models 1 and 2 to classify the stages of DR using both balanced and imbalanced datasets. The proposed two models outperform other single deep learning architectures in terms of accuracy, such as DenseNet-121, ResNet50, Inception-V3, and Dense169, which indicates that ensemble technique can strengthen the capability of classifying model. Model 2 yields higher accuracy with 80.36% than Model 1 does with 78.13% on imbalanced dataset, which indicates that diversity of base classifiers used for ensemble framework is the key factor to high accuracy of ensemble classifying model. Moreover, the confusion matrices of Models 1 and 2 with the balanced and imbalanced datasets have shown equalization of training dataset which makes the classifying model more stable.

In the future, we intend to extend Kaggle dataset by adding fundus images of the same patient during a long period in collaboration with doctors and, oreover, training specific models for specific stages to increase the accuracy of early stages.

## Figures and Tables

**Algorithm 1:**
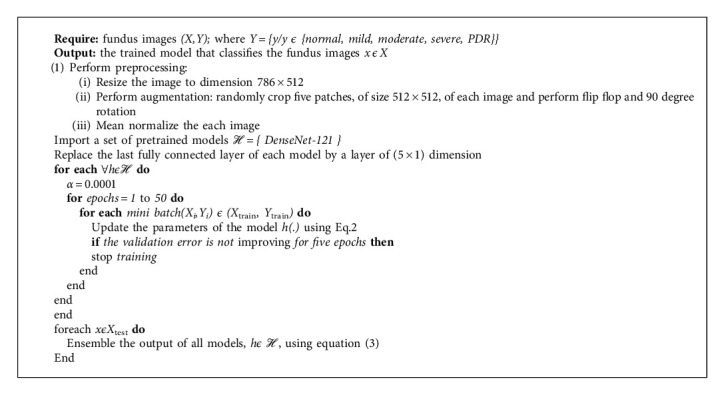
The proposed algorithm.

**Algorithm 2:**
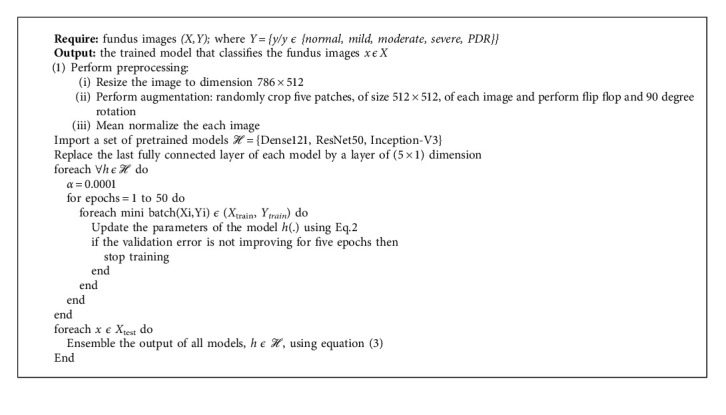
The proposed algorithm.

**Table 1:** Dataset samples in each dataset.

Dataset/class	0	1	2	3	4	Total
Original	25810	2443	5292	873	708	**35126**
Balanced	25810	25810	25810	25810	25810	**129050**
Training	16518	16518	16518	16518	16518	**82590**
Dataset_1	5506	5506	5506	5506	5506	**27530**
Dataset_2	5506	5506	5506	5506	5506	**27530**
Dataset_3	5506	5506	5506	5506	5506	**27530**
Test	5160	5160	5160	5160	5160	**25800**
Valid	4132	4132	4132	4132	4132	**20660**

**Table 2:** Test dataset samples in both balanced and imbalanced datasets.

Class dataset	0 (normal)	1 (mild)	2 (moderate)	3 (severe)	4 (PDR)	Total
Imbalanced	4119	391	845	140	113	**5608**
Balanced	5160	5160	5160	5160	5160	**25800**

**Table 3:** Model 1 class-wise result of balanced and imbalanced dataset.

Dataset	Balanced			Imbalanced		
Class	Recall	Precision	Specificity	Recall	Precision	Specificity
Class 0	0.974811	0.620958	0.776313	0.971595	0.810614	0.288973
Class 1	0.561047	0.629622	0.882527	0.02046	0.117647	0.986468
Class 2	0.430233	0.394597	0.798163	0.298225	0.604317	0.961583
Class 3	0.472868	0.648247	0.909147	0.457143	0.561404	0.988553
Class 4	0.60124	0.835938	0.953846	0.495575	0.777778	0.996315
Accuracy	60.80			78.13		

**Table 4:** Model 2 class-wise result of balanced and imbalanced dataset.

Dataset	Balanced			Imbalanced		
Class	Recall	Precision	Specificity	Recall	Precision	Specificity
Class 0	0.980236	0.609959	0.767048319	0.981063	0.827735	0.356542
Class 1	0.510659	0.644727	0.900055	0.005115	0.060606	0.993166
Class 2	0.437597	0.408023	0.804172	0.400000	0.686992	0.964377
Class 3	0.539147	0.63458	0.889753	0.485714	0.519084	0.986006
Class 4	0.576826	0.849843	0.960332	0.513274	0.828571	0.997310
Accuracy	60.89			80.36		

**Table 5:** Comparison of models.

Models	Imbalance acc.	Balance acc.	Recall	Specificity
Batch_size_2	77.00	60.44	48.36	85.40
Batch_size_3	77.40	61.62	49.87	84.98
Batch_size_4	77.78	60.41	46.33	85.48
Model 1	78.13	60.80	44.85	84.43
DenseNet-121	78.08	—	49.80	85.93
ResNet50	77.28	—	45.14	84.8
Inception-V3	71.25	—	48.17	84.54
Xception	74.41	—	46.26	84.61
Dense169	73.91	—	47.58	84.85
Pratt et al.'s [20] model	75.00	—	30.00	95.00
Model 2	**80.36**	**60.89**	**47.70**	**85.94**

## Data Availability

The public dataset, Kaggle, is used and it can be accessed from https://www.kaggle.com/.
